# Mitogenomics of the zoonotic parasite *Echinostoma miyagawai* and insights into the evolution of tandem repeat regions within the mitochondrial non-coding control region

**DOI:** 10.1017/S0031182024001422

**Published:** 2024-12

**Authors:** Linh Thi Khanh Pham, Dong Van Quyen, Weerachai Saijuntha, Huong Thi Thanh Doan, Thanh Hoa Le, Scott P. Lawton

**Affiliations:** 1 Immunology Department, Institute of Biotechnology (IBT), Vietnam Academy of Science and Technology (VAST), 18. Hoang Quoc Viet Rd., Cau Giay, Hanoi, Vietnam; 2 University of Science and Technology of Hanoi (USTH), Vietnam Academy of Science and Technology, 18 Hoang Quoc Viet, Cau Giay, Hanoi, Vietnam; 3 Molecular Microbiology Department, Institute of Biotechnology (IBT), Vietnam Academy of Science and Technology (VAST). 18. Hoang Quoc Viet Rd., Cau Giay, Hanoi, Vietnam; 4 Faculty of Medicine, Mahasarakham University, Mahasarakham 44000, Thailand; 5 Graduate University of Science and Technology (GUST), Vietnam Academy of Science and Technology (VAST), 18. Hoang Quoc Viet Rd., Cau Giay, Hanoi, Vietnam; 6 Centre for Epidemiology & Planetary Health, School of Veterinary Medicine & Biosciences, Scotland's Rural College, Inverness Campus, Inverness IV2 5NA, UK

**Keywords:** Echinostomatids, genome, mitochondria, palindrome, tandem repeat units

## Abstract

*Echinostoma miyagawai* is a cosmopolitan parasite within the Echinostomatidae and is a cause of human echinostomiasis. Species within the family have been a challenge to disentangle with *E. miyagawai* being synonyms of several other *Echinostoma* species. However, complete mitochondrial genomes have been shown to be vital in distinguishing echinostomatid species, but detailed comparisons of not only gene content but also structural features have been limited. Using long range sequencing techniques, the complete mitochondrial genome of *E. miyagawai* was sequenced and compared to other members of Echinostomatidae. In total 12 protein coding genes, 2 ribosomal RNA genes and 22 transfer RNA genes were identified, as was an extensive noncoding control region (CR), consisting of 2 types of multiple tandem repeat units. Phylogenetic analyses of complete mitochondrial genomes corresponded to previous studies on single mitochondrial genes and nuclear ribosomal nuclear markers confirmed *E. miyagawai* to be within in the ‘*Echinostoma revolutum’* group. The tandem repeat units found in the CR contained promoter sequences containing domains typical of initiation sites for replication and transcription as well as several palindromic regions which were shared between echinostomatid species. The study illustrates not only the utility complete mitogenomes in disentangling the relationship between these parasite species, but also provides some insight into the potential adaptations and other evolutionary processes that may govern the divergence of mitochondrial genomes for the first time in echinostomatids.

## Graphical abstract

**Figure:**
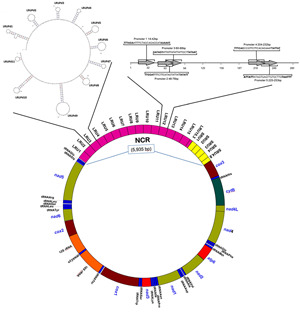

## Introduction

According to the world health organisation (Foodborne trematode infections (who.int)) infections with zoonotic food borne trematodes (FBT) result in an estimated 200 000 illnesses, 2 million life years lost to disability with up to 7000 deaths each year. Predominantly with foci in Asia there are several genera of FBT that cause disease in humans with *Clonorchis*, *Opisthorchis, Paragonimus* and *Fasciola* being the most important. However, in recent years in the Far East and Southeast Asia neglected species within the genus *Echinostoma* have been shown to have a significant impact on public health (Toledo and Esteban, 2016; Jung *et al*., 2024). Human infections of echinostomiasis occur after eating raw or insufficiently cooked mollusc, fish, crustaceans and amphibians harbouring metacercariae. The current incidence and extent of human echinostomiasis remains unknown, with most cases reported from Asia, occurring in endemic foci where the presence of snail intermediate hosts and natural definitive hosts, particularly waterfowl, coexist with the human practice of eating under cooked shellfish and aquatic vertebrates (Toledo and Esteban, 2016; Chai, 2019; Toledo *et al*., 2022).

In recent years mitochondrial gene markers and full mitogenomes have been shown to be highly effective in distinguishing between trematode species, particularly within the Echinostomatoidae (Wey-Fabrizius *et al*., 2013; Bernt *et al*., 2013*a*; Solà *et al*., 2015). *Echinostoma miyagawai* Ishii, 1932, is a suspected leading cause of human echinostomiasis, but is challenging to distinguish from other echinostome species and has historically been considered a synonym of other species including the highly zoonotic *Echinostoma revolutum* (Wu, 2013; Fu *et al*., 2019; Li *et al*., 2019; Le *et al*., 2020; Pham *et al*., 2022). The cryptic nature of *Echinostoma miyagawai* has caused issues in species identification and ultimately impacted upon the understanding of the full epidemiological importance of the parasites, but the application of mitogenomes and associated markers have supported the division of *E. miyagawai* isolates from Eurasia and Australia, as well as defining distinct lineages of *E. revolutum* from Eurasia and the Americas (Georgieva *et al*., 2013, 2014; Nagataki *et al*., 2015; Tkach *et al*., 2016). However, despite the continued revision of the phylogenetic relationships among the echinostomatid taxa, molecular studies indicate a need to reinvestigate and reconsider the relationship between echinostome species, not only based on nucleotide subsitutions but also structural landscape differences across mitogenomes. More recently there has been a substantial increase in the interest in the non-coding regions (NCR) and their repetitive elements as has been studied in trematode species from the families Fasciolidae, Paragonimidae, Brachycladiidae, Diplostomidae and Schistosomatidae (Biswal *et al*., 2013; 2014; Brabec *et al*., 2015; Briscoe *et al*., 2016; Kinkar *et al*., 2019; 2020; Oey *et al*., 2019*a*, 2019*b*; Jones *et al*., 2020; Le *et al*., 2020; 2022; Gacad *et al*., 2023). The NCR is commonly referred to as the control region (CR) as it is crucial in the regulation of gene functionality owing to the occurrence of promoter sites for transcription factor binding, and the origin of mtDNA replication (Tyagi *et al*., 2020). Across many animal phyla, the CR is the most polymorphic region of the mitochondrial genome. As a consequence of uniparental inheritance, a lack of effective recombination, and reduced proofreading it has a higher rate of evolution relative to the genes within the mitochondrial genome. In turn this can lead to an accumulation of highly repetitive sequences, which can increase the overall length of the mitochondrial genome (Howe and Denver, 2008). In trematodes the long NCR region and its function has not been explored in detail and only recently using long read sequencing approaches have these regions become not only accessible but quantifiable (Kinkar *et al*., 2020). However, there is a deficit in detailed comparisons between echinostomatid species and little known about the variation in length caused by NCR repeat elements of the mitogenomes.

Therefore, by applying long read next generation sequencing approaches the aim of this current study was to sequence through the whole mitogemome of *Echinostoma miyagawai* to characterize the NCR, capturing the multiple repeat units, representing for the first time the longest and most complete mitogenome among echinostomatids. The mitogenomic genes were comparatively characterized and used for taxonomic and phylogenetic studies of the Echinostomatidae.

## Materials and methods

### Parasite samples and DNA extraction

Adult flukes of Echinostoma miyagawai were collected from the intestines of naturally infected domestic ducks slaughtered in abattoirs in Roit Et province, Thailand. After washing in physiological saline the flukes were morphologically examined based on the *Echinostoma* characteristics and the presence of 37 collar-spines around the head for the ‘*revolutum*’ group and the keys to the morphology of the echinostomes as previously described (Georgieva *et al*., 2014; Faltýnková *et al*., 2015; Chai., 2019). The *E. miyagawai* samples used in this study (named: RED-11) were molecularly identified using *nad*1 and *cox*1 markers and belonged to the Eurasian lineage of the ‘*Echinostoma revolutum*’ complex (Nagataki *et al*., 2015).

Total genomic DNA was extracted from a single adult worm using the GeneJET™ Genomic DNA Purification Kit (Thermo Fisher Scientific Inc., MA, USA) according to the manufacturer's instructions. The genomic DNA was eluted in 50 μL of the elution buffer and kept at –20°C until use and the DNA content was quantified using a *NANODROP*® ND-*1000* UV-Vis Spectrophotometer. For mitogenomic DNA enrichment, a working concentration (50 ng μL^−1^) was prepared and an amount of 2 μL was used in each long-range PCR (LPCR) in a 50 μL reaction volume.

### Targeted enrichment of the mitogenome by long range polymerase chain reaction

Eight primer pairs, including trematode-, Echinostomatidae-universal, and *E. miyagawai* specific primers were designed and used for amplifying the whole mtDNA of *E. miyagawai* in 8 overlapping fragments using the long-range PCRs. Among these, 2 amplicons, obtained by primer pairs EMY5F-EMY3R* and EHC5F-EHC3R*, were used for comparative validation of the length of the NCR (Supplementary Table 1). Long-range PCRs were performed in a 50 μL volume in a MJ PTC-100 Thermal Cycler, with each reaction containing 25 μL 2X LongAmp Master Mix (New England Biolabs, Ipswich, MA, USA), 2 μL each primer (10 pmol μL^−1^), 2 μL of template DNA and 19 μL DEPC-water. The LPCRs were conducted with an initial denaturation at 94°C for 1 min, followed by 30 cycles, each consisting of a denaturation step for 30 s at 94°C, an annealing/extension step at 50°C for 30 s, extension at 65°C for 8 min, and a final extension at 65°C for 10 min. The amplificatoin products (10 μL of each) were examined on a 1% agarose gel, stained with ethidium bromide and visualized under UV light (Wealtec, Sparks, NV, USA).

The dsDNA products were purified using the GeneJET PCR Purification Kit (Thermo Fisher Scientific, USA) and the amplicon length was verified *via* 1.5% agarose gel electrophoresis. Resultant amplicons from the coding mtDNA and 2 from the NCR were pooled for NGS. The complete mitogenome of *E. miyagawai* was sequenced using the PacBio SEQUEL system (https://www.pacb.com/) with a targeted long-read sequencing approach at the PacBio facility at the Institute of Biotechnology (Hanoi, Vietnam). The dsDNA products of each sample from 8 overlapping amplicons were pooled and purified with AMPure XP beads (Pacific Biosciences, Menlo Park, CA, USA). Input dsDNA was quantified using the Qubit fluorometer 3.0 and Qubit dsDNA HS Assay reagents (Thermo Fisher Scientific, Waltham, MA, USA). The SMRTbell Libraries were prepared using the Express Template Prep Kit 2.0 with multiplexing amplicons protocol with low DNA input (100 ng) (Pacific Biosciences, Menlo Park, CA, USA) for sequencing on the PacBio SEQUEL system according to the manufacturer's instructions. The SMRTbell templates were purified once with 1.2 volumes of AMPure PB beads and the size and amount of the library were checked again using the Bioanalyzer 2100 system (Agilent, CA, USA) and the Qubit fluorometer 3.0 with Qubit™ dsDNA HS Assay reagents, respectively. The libraries of all amplicons were then pooled before long-read sequencing.

### Sequencing and de novo assembly

The pooled library was bound to polymerase using the Sequel Binding and the Internal Control Kit 3.0 (Pacific Biosciences, Menlo Park, CA, USA) and purified using Ampure PB beads. The DNA Control Complex 3.0 and the Internal Control Kit 3.0 from Sequel Binding and Internal Ctrl Kit 3.0 were used to control the sequencing procedure. The final library was loaded onto Sample Plate (Pacific Biosciences, Menlo Park, CA, USA). The run design was created by the Sample Setup software included in the SMRTLink portal v5.1 version 9.0 with an insert size of 1200 base pairs (bp). The sequencing signals were processed, evaluated, and converted into raw data by the Primary Analysis Computer server. All data was automatically transferred to the Secondary Analysis Server system *via* the intranet. High quality sequence data was proofread and generated by PacBio's circular consensus sequencing (CCS), then *de novo* assembled using Canu software v2.0 (Koren *et al*., 2017), and the quality of the assembly was checked by using Quast software v5.0.2 (Gurevich *et al*., 2013).

### Annotation of mitogenome and gene characterization

Twelve protein-coding genes (PCG) were identified by comparative alignment with the available mitogenomes of the *E. miyagawai* strains and other echinostomatid species in GenBank or recently reported (Li *et al*., 2019; Fu *et al*., 2019; Le *et al*., 2020; Pham *et al*., 2022) (Supplementary Table 2). ATG/GTG as the start and TAA/TAG as the stop codons were used to define individual PCG gene boundaries. The ‘*echinoderm and flatworm mitochondrial genetic code*’ (translation Table 9 in GenBank) was used for the translation of the PCGs. Transfer RNA genes were identified using the tRNAscan-s.e. 1.2.1 program (www.genetics.wustl.edu/eddy/tRNAscan-s.e./) (Lowe and Chan, 2016), ARWEN (http://mbio-serv2.mbioekol.lu.se/ARWEN/) (Laslett and Canback, 2008), and the MITOS Alpha version (http://mitos.bioinf.uni-leipzig.de/index.py) (Bernt *et al*., 2013*b*). Final sequences and secondary structures were based on comparisons using all these programs.

Gene nucleotide comparison for divergence rate (%) between *E. myiagawai* strain RED11 and other echinostomatids were estimated based on the alignment of individual genes, PCGs, and mitochondrial ribosomal genes (MRGs) using MAFFT v7.407 (Katoh and Standley, 2013), curated using BMGE v1.12 (Criscuolo and Gribaldo, 2010) in NGPhylogeny (available at https://ngphylogeny.fr) (Lemoine *et al*., 2019), and MEGA 11 (https://www.megasoftware.net/) (Tamura *et al*., 2021) for percentage calculation. The nucleotide composition for concatenated PCGs, MRGs, and the mtDNA coding region (5′-*cox*3 to *nad*5-3′, designated as mtDNA*) was analyzed and calculated with MEGA 11. Skew values (ranging from − 1 to + 1) were determined by calculating the percentage of AT and GC nucleotide usage using Perna and Kocher's (1995) formula: AT skew = (A − T)/(A + T), and GC skew = (G − C)/(G + C), where the letters represent the absolute usage of the corresponding nucleotides in the sequences.

### Phylogenetic reconstruction

Concatenated aligned amino-acid sequences of 12 PCGs from 12 species from the Echinostomatidae were used for phylogenetic reconstruction using KF543342 *Fasciola gigantica* and AF216697 *Fasciola hepatica* from the sister family Fasciolidae as an out group (listed in Supplementary Table 2). In addition to 3 strains of *E. miyagawai* (the RED-11 of Thailand and the Hunan and HLJ strains of China), the 10 available echinostomatid mtDNA sequences from 11 species of the family Echinostomatidae were included. Among these were 3 representatives of the 37 collar-spined ‘revolutum’ group (*Echinostoma caproni* from Egypt, *E. revolutum* from Thailand, and *E. paraensei*), 2 artyfechinostomid species (*Artyfechinostomum malayanum*, Thailand and *Artyfechinostomum sufrartyfex*, India), 1 *Hypoderaeum conoideum* (China), 1 *Echinoparyphium aconiatum* (Russia), and 3 genus- or family-level identified species (Echinostomatidae sp. CA-2021 isolate PE4, United States (MK264774); *Echinostoma* sp. isolate JM-2019, China (MH212284); Echinostomatidae sp. MSB para 30070, isolate A_19, United States (MN822299)) (Yang *et al*., 2015; Fu *et al*., 2019; Li *et al*., 2019; Le *et al*., 2020; Pham *et al*., 2022; Gacad *et al*., 2023). One echinostomatid species, the GD strain (China, MN116706) (Ran *et al*., 2020), was reported as ‘*Echinostoma revolutum*’ but due to the lack of strong *Echinostoma* generic evidence, it was listed in the cryptic genus as an unidentified taxon within the Echinostomatidae (Pham *et al*., 2022), was also included in the analysis.

The concatenated protein-coding nucleotide sequences were imported into GENEDOC 2.7 (available at: https://softdeluxe.com/GeneDoc-180568/download/) and translated using the ‘*Echinoderm and flatworm mitochondrial genetic code’* (Translation Table 9) in GenBank. The PhyML software package in the NGPhyogeny (at https://ngphylogeny.fr), was used to perform phylogenetic analyses. The input consisting of concatenated amino acid sequences in FASTA format was uploaded and aligned using MAFFT v7.407, then curated using BMGE v1.12, and tree inferred by PhyML v3.3.1 using maximum likelihood with 1000 bootstrap replicates (Guindon *et al*., 2010), with the best-quality final sequence block of 2949–3107 amino acids (aa). The resulting Newick tree (.nwk) (Junier and Zdobnov, 2010) was visualized using FigTree 1.4.4 program (Rambaut, 2018). Phylogenetic analysis and tree reconstruction, including the outgroup sequence, were also completed using the maximum likelihood (ML) analysis in the MEGA 11 program (Tamura *et al*., 2021). The substitution model with the best score, according to the Bayesian information criterion, was the Jones, Taylor, and Thornton + F + G + I model (JTT + F + G + I), with residue frequencies estimated from the data ( + F), rate variation along the length of the alignment ( + G), and allowing for a proportion of invariant sites ( + I) (Tamura *et al*., 2021).

### Identification of the non-coding region and its structural features

The NCR was identified as a region between the 3′ end of the tRNA^Glu^ and the 5′end of the cox3 gene. Repeat sequences were detected in the NCR using the Tandem Repeats Finder v3.01 (Benson, 1999). The circular map and gene abbreviations on the map were generated by using the GenomeVx v2.0 drawing tool (http://conantlab.org/GenomeVx/) (Conant and Wolfe, 2008). The NCR of the mitochondrial genome is involved in the regulation of transcription of the mitogenes, and as such a substantial amount of the sequence content is repetitive and corresponds to promoter regions. To identify putative promotor regions in the long repeat units (LRU) and the short repeat units (SRU) sequences they were submitted to SAPPHIRE.CNN SAPPHIRE (kuleuven.be), a web based server that employs neural network algorithms to predict promoter regions in prokaryotic sequences (Coppens and Lavigne, 2020). Mitochondrial repeat units are known to have a high number of palindromic sequences in other invertebrates and in order to identify the occurrence of such sequences the *E. miyagawai* LRU and SRU sequences were submitted to the UNAFold Web Server (www.unafold.org) using default settings (Zuker, 2003). This analysis predicts folding sites within a DNA sequence based on experimental values on melting points of nucleotides and their combinations within a DNA loop domain to predict the most likely complementary sequences that could create a palindromic hairpin motifs.

## Results

### Echinostoma miyagawai mitogenomic gene order

**Figure 1.**
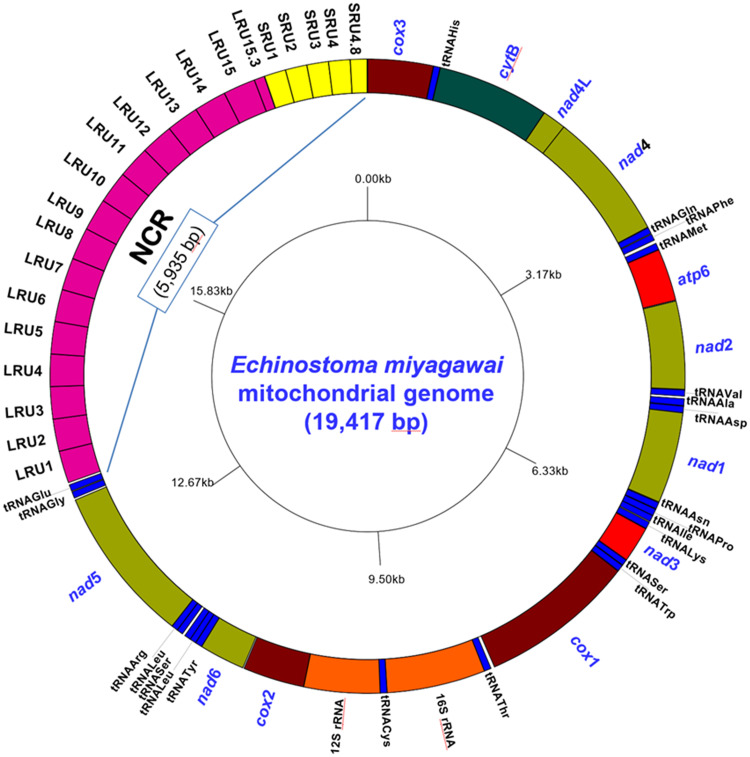
A schematic drawing of a circular map of the mitogenome of *Echinostoma miyagawai* (GenBank: OP326312). The circular mtDNA map was created using GenomeVx v2.0 (http://conantlab.org/GenomeVx/). The protein-coding (PCGs) and mitoribosomal large and small subunit genes (MRGs) are abbreviated as in our previous publications (Le *et al*., 2020, 2022). The transferRNA genes (tRNAs) are marked with three-letter amino acid abbreviations (see Table 1). The non-coding region (NCR, 5,935 bp) contains tandem repeat units and is located between tRNA^Gly^ and *cox*3 with 15.3 LRUs (long, LRU1–15.3) and 4.8 SRUs (short repeat units, SRU1–4.8).

The whole mitogenome of *Echinostoma miyagawai*, strain RED11 from Thailand, is 19 417 bp in length (GenBank accession no. OP326312) (Fig. 1; Supplementary Table 3), which is the longest complete mtDNA to be sequenced so far among the echinostomatid species. The circular mtDNA molecule is comprised of 12 PCGs (*cox*1 − 3, *cob*, *nad*1 − 6, *nad*4L, *atp*6), 2 MRGs (16S or *rrn*L and 12S or *rrn*S), 22 tRNAs or *trn*, and an NCR that possesses long and short tandem repeats. The genome was assembled using 2 major over lapping contigs of 2954 bp and 7029 bp which formed an almost complete circular genome of 19 083 bp. Several small gaps within the coding region were found in the first contig upon sequence comparisons with other echinostomatid species and subsequently filled by conventional Sanger sequencing. The second contig perfectly matched the expected genes from the 3′ end of *NAD*5 and the 5′ end of *CYT*B. This fragment also contained the tRNA^Gly^, tRNA^Glu^, tRNA^His^, and *cox*3 genes and entirely bridged the tandem repeat non-coding region (5,935 bp).

The *E. miyagawai* mitogenome length was much longer than that in the other members of the Echinostomatidae, e.g., *Echinostoma revolutum* (17 030 bp, strain MSD15, Thailand) (Le *et al*., 2020), *Artyfechinostomum malayanum* (17 175 bp, strain EMI3, Thailand) (Pham *et al*., 2022), *Artyfechinostomum sufrartyfex* (14 567 bp, strain Shillong, India; GenBank: KY548763), *Echinostoma caproni* (14 150 bp, strain SAMEA, Egypt; GenBank: AP017706), *Hypoderaeum conoideum* (14 180 bp, strain Hubei, China) (Yang *et al*., 2015), *Echinoparyphium aconiatum* (14 865 bp, strain Chany, Russia) (Gacad *et al*., 2023), and several echinostomatid species to be reported to date, likely as a result of the mitogenomes of these species being incomplete. Similarly, in comparison between other echinostomatid species, and geographical isolates of *E. miyagawai*, the two Chinese *E. miyagawai* strains (Hunan and HLJ) have mtDNA much shorter (14 468 bp and 14 410 bp, respectively) and seemed to be truncated by conventional sequencing (Fu *et al*., 2019; Li *et al*., 2019) than the current studied Thai isolate (Supplementary Table 3).

### Mitogenomic sequence and phylogenetic comparisons within *Echinostoma miyagawai* and between other echinostomatid species

At the nucleotide level, between *E. miyagawai* and the other echinostomatids, the *nad*6 gene showed the highest divergence over 64.68%, with some genes reaching 71.88 to 75.48% between species. A lower divergence rate is seen for all genes between the *E. miyagawai* RED11 strain and others in the ‘*revolutum*’ group (*E. caproni* SAMEA strain, *E*. *paraensei*, and *E. revolutum* MSD15 strain) (Table 1; Supplementary Table 3). Similarly, the *cyt*B and *cox*1 genes showed the lowest divergence at 13.67% to 27.08% for *cyt*B; 14.14% to 28.61% for *cox*1. The PCGs and the MRGs showed almost the same moderate divergence rate for interspecific variation between *E. miyagawai* and other echinostomatids (Table 1; Supplementary Table 3). The three *E. miyagawai* geographical isolates showed low levels of divergence at less than 1% for all genes except for *cox*3 with a divergence of 1.57% and *nad*1 1.12% between the RED11 and HLJ isolates. The *nad*4 also showed a higher divergence between the RED11 and Hunan isolates at 1.18% (Table 1). This nucleotide usage of *E. miyagawai* does not vary considerably across the *Echinostoma* genus but is different in other echinostomatids with lower A + T such as in *Artyfechinostomum malayanum*, *A. sufrartyfex*, *Hypoderaeum conoideum*. Except for *Echinoparyphium aconiatum*, Echinostomatidae sp. MSB para 30070, and *E. revolutum* isolate MSD15, which had the AT-skew of low negative (−0.414 to –0.432/PCGs and −0.357 to −0.379/mtDNA*), the other echinostomatids exhibit highly negative values (−0.477 to −0.483/PCGs and −0.385 to −0.420/mtDNA*), indicating that T was more frequently used than A. The data indicated that the pattern of base usage for all PCGs, MRGs, and mtDNA*s in all 14 strains/species is T > A > G > C, giving the AT-skew negative and the GC-skew positive (Supplementary Table 4).

**Table 1.** Nucleotide comparison for divergence rate (%) of individual and concatenated protein-coding (PCGs) and mitoribosomal genes (MRGs) between *Echinostoma miyagawai* (isolate RED11, Thailand) and members of the family Echinostomatidae (Platyhelminthes: Echinostomata)

Species	*Echinostoma miyagawai* (Emiy-RED11-TH)															
	Protein-coding genes (PCGs)													Mitoribosomal genes (MRGs)		
	*atp*6	*cox*1	*cox*2	*cox*3	*cyt*b	*nad*1	*nad*2	*nad*3	*nad*4L	*nad*4	*nad*5	*nad*6	PCGs	*rrn*L	*rrn*S	MRGss
Amala-EMI3-TH	35.24	23.74	25.24	25.68	24.42	30.50	44.25	30.35	27.51	48.95	35.19	66.77	29.89	28.47	38.92	30.66
Asufr-Shillong-IN	34.80	23.96	25.79	25.71	24.60	31.29	45.31	29.78	26.77	48.24	35.05	67.16	30.01	27.99	38.92	30.34
Eacon-Chany-RU	38.61	28.61	37.09	24.65	27.08	30.64	47.14	36.24	29.32	48.48	36.93	**75**.**48**	32.49	28.99	35.25	30.03
Ecapr-SAMEA-EG	19.27	15.10	15.90	15.01	14.85	15.79	20.95	16.40	19.56	22.28	20.54	30.65	17.36	13.66	11.66	12.98
EchCA2021-PE4-US	48.30	26.93	46.04	28.99	25.81	30.87	45.75	30.24	32.36	44.97	39.36	**71**.**88**	32.95	29.09	36.96	30.11
Ech-JM2019-CN	38.73	23.16	21.70	23.45	24.49	26.77	40.16	34.16	30.79	41.07	33.66	65.30	28.39	27.31	30.52	27.59
EchMSB-A19-US	42.91	27.29	48.82	27.46	24.90	32.05	44.53	36.63	27.31	46.61	40.36	69.19	33.20	27.00	38.40	29.16
Emiya-HLJ-CN	00.78	00.39	00.33	**01**.**57**	00.45	**01**.**12**	00.81	00.85	00.73	00.78	00.90	00.31	**00**.**69**	00.31	00.13	00.23
Emiya-Hunan-CN	00.77	00.26	00.33	00.47	00.36	00.89	00.70	00.00	00.37	**01**.**18**	00.58	00.62	**00**.**58**	00.21	00.27	00.29
Epara	24.27	15.71	16.87	13.39	14.31	17.39	19.51	16.88	20.82	23.53	16.97	34.18	17.29	11.22	14.02	12.28
Erevo-GD-CN	39.55	23.61	22.00	22.97	24.24	26.85	40.10	34.60	29.98	41.76	34.12	64.68	28.57	27.16	30.59	27.49
Erevo-MSD15-TH	19.29	14.14	**13**.**07**	13.75	13.67	15.10	18.69	18.60	**10**.**29**	21.04	18.68	33.36	**16**.**09**	12.83	11.84	**12**.**39**
Hcono-Hubei-CN	46.16	27.99	43.06	28.96	26.45	32.55	49.70	32.04	25.29	43.79	41.11	**73**.**96**	33.42	31.21	39.93	32.44

*Note*: Amala-EMI3-TH: *Artyfechinostomum malayanum* isolate EMI3, Thailand (OK509083); Asufr-Shillong-IN: *A. sufrartyfex* isolate Shillong, India (KY548763); Eacon-Chany-RU: *Echinoparyphium aconiatum* isolate Chany, Russia (ON644993); Ecapr-SAMEA-EG: *E. caproni* isolate SAMEA, Egypt (AP017706); EchCA2021-PE4-US: Echinostomatidae sp. CA-2021 isolate PE4, United States (MK264774); Ech-JM2019-CN: *Echinostoma* sp. isolate JM-2019, China (MH212284); EchMSB-A19-US: Echinostomatidae sp. MSB para 30070, isolate A_19, United States (MN822299); Emiya-HLJ-CN: *E. miyagawai* isolate Heilongjiang, China (MH393928); Emiya-Hunan-CN: *Echinostoma miyagawai* isolate Hunan, China (MN116740); Epara: *E. paraensei* (KT008005); Erevo-GD-CN: *E. revolutum* isolate Guangdong, China (MN116706); Erevo-MSD15-TH: *E. revolutum* isolate MSD15, Thailand (MN496162); Hcono-Hubei-CN: *Hypoderaeum conoideum* isolate Hubei, China (KM111525). The inter-specific divergence (%) between *E. miyagawai* isolate RED11 (Thailand) and other echinostomatids for the most conserved *cyt*B and *cox*2, and for the most divergent *nad*6 genes are highlighted. The highest value in *cox*3, *nad*1, and *nad*4, and the average value in PCGs for the intra-specific divergence within the *E. miyagawai* strains, are background shaded and boxed. The highest inter-specific divergence between *E. miyagawai* and other echinostomatids in *nad*6 are boxed. *Echinostoma revolutum* (MSD15 strain) is the most related species (background shaded) and the lowest divergence between this species and *E. miyagawai* in *cox*2, *nad*4L, PCGs, and MRGs is bolded.

**Figure 2.**
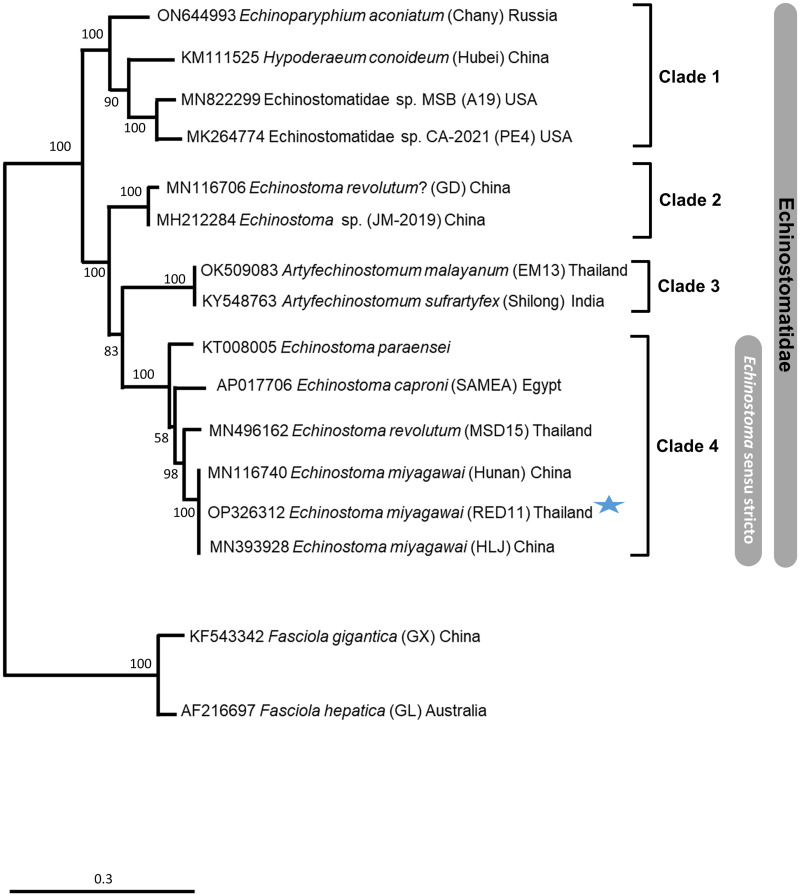
A maximum-likelihood phylogenetic tree showing the position of *Echinostoma miyagawai* and the phylogenetic relationships among the taxa within the family Echinostomatidae. Where the blue star represents the complete mitochondrial genome generated in this study. The phylogeny was constructed using the concatenated protein sequences produce by the twelve protein coding genes across the mitochondrial genome under the conditions of the JTT + F + G + I model. Bootstrap values are provided as nodal supports, and the scale bar represents substitutions per amino-acid positions.

To assess the mitophylogenetic and taxonomic relationships between *E. miyagawai* and other taxa within the family Echinostomatidae, a comprehensive phylogenetic analysis was performed on the alignment of the amino-acid sequences of 12 PCGs for 12 species of 9 and 3 isolates of *E. miyagawai* from China (Hunan, HLJ) and the RED11 isolate from Thailand generated in this study. The ML phylogeny split into 4 well defined clades with bootstrap support values 83–100 (Fig. 2). Clade 1 being the most basal contained *Echinoparyphium aconiatum*, *Hypoderaeum conoideum*, and two cryptic species of Echinostomatidae (MSB_(A19)/MN822299 and CA-2021-(PE4)/MK264774) from the USA (Fig. 2). Clades 2–4 contained all member of the genus *Echinostoma,* with species within the *Artyfechinostomum* genus (Clade 3) being phylogenetically bracketed between *Echinostoma* species (clades 2 and 4). Clade 2 contained two cryptic *Echinostoma* species from China and, Clade 4 contained all species considered to be *Echinostoma* sensu stricto where *E. paraensei* appeared to be basal and *E. revolutum* (MSD15), *E. caproni* formed sister groups to the three *E. miyagawai* strains which formed a one subcluster supported with 100 bootstrap values (Fig. 2).

### The structural features and evolution of the non-coding control region of the *Echinostoma miyagawai* mitochondrial genome

#### The structural features of the repetitive non-coding region

Using the long-reading PacBio system, the complete NCR in the mitogenome of the *E. miyagawai* RED11 strain was successfully obtained measuring 5,935 bp, containing 2 types of tandem repeat units referred to as the long repeat unit, the LRU, and short repeat unit, the SRUs, and was flanked by tRNA^Glu^ (*trn*E) and the *cox*3 gene. The relatively long NCR (near 6.0 kb) was divided into 2 subregions: the first subregion contained 15 identical LRUs of 319 bp and a partial one of 102 bp, and the second contained 4 SRUs of 213 bp/each and a partial one of 165 bp. Only three nucleotides (TAA, position: 18393–18395) connected the 3′ end of the last LRU15.3 and the 5′ end of the first SRU1 (Fig. 1; Table 2). The identical LRUs were present in the NCR of the Chinese strains as well, but in fewer numbers (2.99 LRUs in the Hunan strain and 2.3 LRUs in the HLJ strain), and no other repeats such as SRUs of the RED11 strain were found in either one (Table 2). The repetitive features in the NCR were not stated in the original analyses by Li *et al*. (2019) and Fu *et al*. (2019).

**Table 2.** An updated summary of the numbers and types of repetitive sequences in the non-coding regions (NCR) of 14 strains and species for the available members of the family Echinostomatidae, indicating high polymorphism and interspecific/intergeneric variation

Species	mtDNA length as reported (bp)	Length of NCR (bp)	Strains and country of collection	Number and size of repeat units (RUs)	Family type of repeats	GenBank accession No	References
*Echinostoma miyagawai*	19 417	5.935	RED11 (Thailand)	15.3 LRUs (319 bp/each) 4.8 SRUs (213 bp/each)	Tandem	OP326312	This study
*Echinostoma miyagawai*	14 460	982	Hunan (China)	2.99 LRUs (319 bp/each) No SRUs	Tandem	MN116740	Fu *et al*. (2019)
*Echinostoma miyagawai*	14 410	931	HLJ (China)	2.3 LRUs (319 bp/each) No SRUs	Tandem	MH393928	Li *et al*. (2019)
*Echinostoma caproni*	14 150	685	SAMEA (Egypt)	None	None	AP017706	GenBank
*Echinostoma paraensei*	20 298*	6,798*	n/a	3.2 RUs (206 bp/each)	Tandem	KT008005	GenBank
*Echinostoma revolutum*	17 030	3,549	MSD15 (Thailand)	7.6 LRUs (317 bp/each) 5.3 SRUs (207 bp/each)	Tandem	MN496162	Le *et al*. (2020)
*Echinostoma revolutum*?	15 714	2,283	GD (China)	3.6 LRUs (245 bp/each) 3.6 SRUs (208 bp/each)	Tandem	MN116706	Ran *et al*. (2020)
*Echinostoma* sp.	15 283	1,877	JM-2019 (China)	5.6 LRUs (245 bp/each) 2.2 SRUs (166 bp/each)	Tandem	MH212284	GenBank
Echinostomatidae sp. CA-2021	14 426	963	PE4 (United States)	None	None	MK264774	GenBank
Echinostomatidae sp. MSB para 30 070	13 985	474	A19 (United States)	None	None	MN822299	GenBank
*Artyfechinostomum malayanum*	17 175	3.622	EMI3 (Thailand)	5.5 LRUs (336 bp/each)7.5 SRUs (207 bp/each)	Tandem	OK509083	Pham *et al*. (2022)
*Artyfechinostomum sufrartyfex*	14 567	1004	Shillong (India)	2.8 RUs (144 bp/each)	Tandem	KY548763	GenBank
*Echinoparyphium aconiatum*	13 377	1,388	Chany (Russia)	4.7 RUs (113 bp/each)	Tandem	ON644993	Gacad *et al*. (2023)
*Hypoderaeum conoideum*	14 180	644	Hubei (China)	None	None	KM111525	Yang *et al*., 2015

*Note*: non-coding region (NCR): sequence between the 3′ terminus of tRNA^Glu^ and the 5′ terminus of *cox*3; *the NCR in *E*. *paraensei* was not fully sequenced, and the number of repeat units in this species is incomplete. RU: repeat unit; LRU: long tandem repeat; SRU: short tandem repeat. These two terms are used when two different sizes are found in the NCR. n/a: not available; none: no repeats were found.

**Figure 3.**
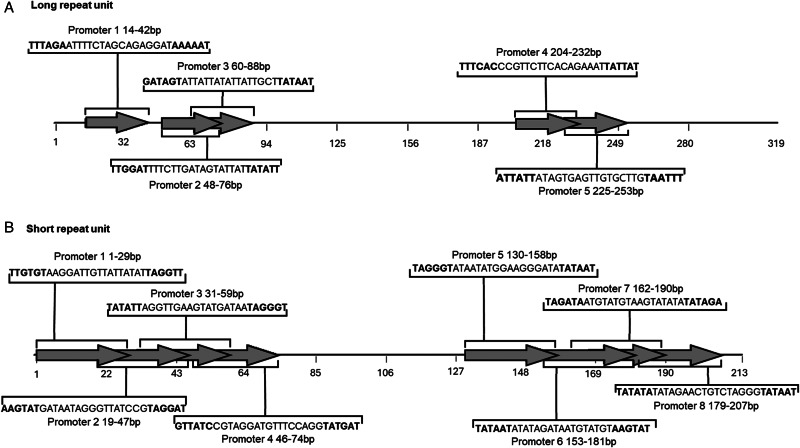
Schematic of the position of predicted promoter regions within the tandem repeat units repeat units of *Echinostoma miyagawai* mitochondrial control region: Where (A) indicates the identification of the five putative promoters within the long repeat unit (LRU) and (B) illustrates the identification of 8 putative promoter regions within the short repeat unit (SRU).

The SAPPHIRE.CNN analyses of LRU and SRU did reveal several putative promoter regions, the likelihood of which were all significant with *P* values <0.01 (Fig. 3). Both repeat units showed there to be clusters of putative promoter sequences predominantly at the start and end of the sequence. In total the LRU had 5 putative promoter sequences between nucleotide position 14–88 and then at the later end of the sequence from 204–253 bp. The SRU had a total of 8 putative promoter sequences, with 4 overlapping promoters identified from nucleotide position 1–74 and then a further 4 promoters identified between 130–207 (Fig. 3). Conserved motifs, including TA(A)n-like sequences, TATA motifs and G(A)nT motifs, typical to the initiation sites for replication and transcription were found across the promoters in the LRU and SRU. However, only promoter 5 in the LRU had indications of a poly T motif at the 5′ end (Fig. 3).

A total of 11 palindromic repeat regions were identified in the long repeat units of *E. miyagawai* with a further 6 identified in the short repeat units (Supplementary Fig. 1). In both cases their appeared to be a high GC or AT content with a bias to either set of nucleotides. There was no consistency in palindromic hairpin length nor the size of the resultant loop domains. However, three large palindromes were identified in the LRU, these were LRUPd1, LRUPd9 and LRUPd10, each of which with had mismatched base pairs or extra nucleotides in the arm of the hairpin region (Table 3). This was also true for the large palindromic sequences identified in the SRU, denoted SRUPd3 and SRUPd5 (Table 3).

**Table 3.** List of palindromic sequences found in the long and short repeat unit in the mitochondrial control region of *Echinostoma miyagawai*.

Repeat unit	Palindrome name	Position	Predicted palindrome sequence
Long repeat unit	LRUPd1	1–34	5′-CTTCTGT(T_2_)TAGA-(N_4_)-TCTAGCAGAGG-3′
	LRUPd2	38–56	5′-AAAAT-(N_9_)-ATTTT-3′
	LRUPd3	74–88	5′-ATTAT-(N_5_)-ATAAT-3′
	LRUPd4	96–114	5′-AAATTTA-(N_4_)-TAAATTT-3′
	LRUPd5	125–143	5′-AGCGA-(N_9_)-TCGCT-3′
	LRUPd6	146–166	5′-CTAAA-(N_11_)-TTTAG-3′
	LRUPd7	168–188	5′-AAAATTTT-(N_5_)-AAAATTTT-3′
	LRUPd8	196–212	5′-TGGG-(N_9_)-CCCG-3′
	LRUPd9	128–25	5′-CACAGAAATTA-(N_19_)-TAATTTTTGTG-3′
	LRUPd10	271–299	5′-CCCCC(T_2_)ACAA-(N_7_)-TTGT(T_2_)GGGGG-3′
	LRUPd11	311–319	5′-AC-(N_5_)-GT-3′
Short repeat unit	SRUPd1	35–42	5′−AT-(N_4_)-AT-3′
	SRUPd2	49–59	5′−ATCC-(N_3_)-GGAT-3′
	SRUPd3	73–95	5′−ATTGGCAT-(N_7_)-ATGGCAAT-3′
	SRUPd4	97–110	5′−CCCCC-(N_4_)-GGGGG-3′
	SRUPd5	150–185	5′−ATATATA(A)TATATA-(N_7_)-TATGTA(TG)TATATAT-3′
	SRUPd6	188–197	5′−AGA-(N_4_)-TCT-3′

Where *N* = the loop domain, nucleotides in green = mismatches, and yellow nucleotides indicate extra uncomplimented nucleotides.

#### Comparisons of non-coding mitochondrial control region

**Figure 4.**
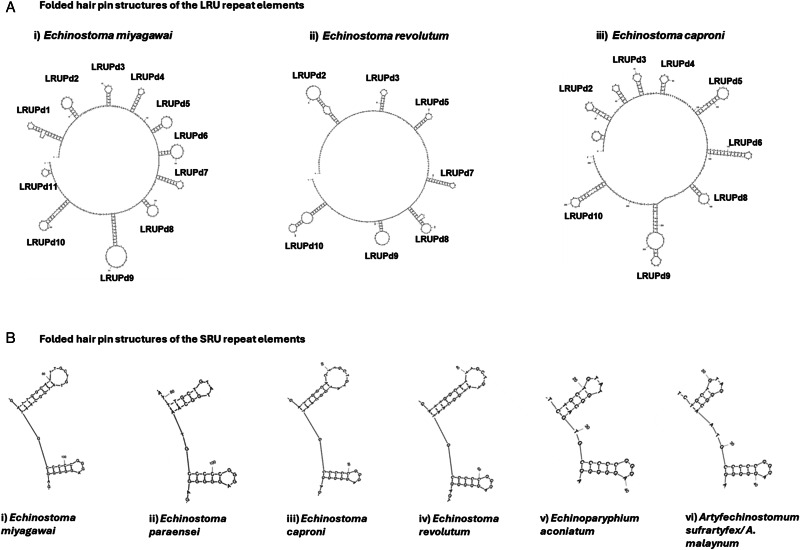
Comparisons of the palindromic hairpin regions tandem repeat units of *Echinostoma miyagawai* mitochondrial control region. Where (A) illustrates the number the palindromic sequences when the full LRU is compared between *E. miyagawai*, *E. revolutum* and *E. Caproni.* (B) illustrates the pattern of conserved palindromes in the SRU across the echinostomatids were SRUPd3 is the upper hairpin and SRUPd4 is the lower hairpin.

Inter species comparisons of the LRU indicated that there were only 5 other species of echinostomatids with available mitochondrial sequences with similarity to the *E. miyagawai* LRU. These species included *Echinostoma revolutum*, *Echinostoma caproni*, *Echinostoma paraensei*, *Artyfechinostomum malayanum*, and an unknown species Echinostomatidae sp. CA-2021, all of which represented by partial sequences. *Echinostoma revolutum* and *E. caproni* had the most complete LRU sequences for comparison with *E. miyagawai*, with *E. revolutum* sharing 7 palindromic hairpin sequences with LRUPd4 and LRUPd6 being absent (Fig. 4A). Also, there were differences in size of palindromes in *E. revolutum* relative to that of *E. miyagawai,* with LRUPd2, LRUPd8 and LRUPd10 being highly extended owing to the occurrence of mismatched or extra base pairs, however the content of the loop domains appeared to be conserved between the 2 species. In comparisons between *E. miyagawai* and *E. caproni* again 7 hairpin palindromes were shared, but unlike in *E. revolutum* LRUPd7 missing (Fig. 4A). In *E. caproni* LEUPd5 and LRUPd9 were substantially extended although the loop domains were homologus to those found in *E. miyagawai* and *E. caproni.* Interestingly, *E. caproni* also had 2 other unique palindromes which were absent in the other 2 species. It is also important to note that the absence of palindromes LRUPd1 and LRUPd11 was the result of missing comparable sequence data from *E. revolutum* and *E. caproni* (Fig. 4A).

In contrast, the interspecies comparisons of SRU revealed 8 other species sharing homology, including *E. paraensei*, *E. caproni*, *E. revolutum*, Echinostomatidae sp. MSB para 30 070 isolate A 19, *Echinoparyphium aconiatum*, *Hypoderaeum conoideum, A. malayanum* and *Artyfechinostomum sufrartyfex.* However, all sequences were partial and comparative palindromic hairpin analyses could only be performed on a 30-base pair region, which was denoted SRUPd3 and SRUPd4 in *E. miyagawai*. These palindromic hairpins were consistent across all species, although SRUPd3 did appear to vary in length between species, SRUPd4 was highly conserved and identical in each of the echinostomatids (Fig. 4B).

## Discussion

With the addition of the newly sequenced mtDNA data it was possible to produce a revised phylogenetic framework to disentangle the taxonomic relationships within and between the Echinostomatidae. The current analysis revealed the Echinostomatidae to be monophyletic, in which the *Echinostoma* species are grouped in a well-supported clade (Clade 4), while the non-*Echinostoma* and the other ‘cryptic’ species appeared as paraphyletic spread across (Clade 1 and 2). Also, two distinct subclusters formed by unidentified species from the Chinese *Echinostoma* spp. and from the American Echinostomatidae spp., which may represent novel genera. A major ‘sister’ relationship was identified across the phylogenetic analyses, the first was between *Echinostoma* and *Artyfechinostomum*, showing good congruence to previous phylogenetic reconstructions based on ribosomal markers (Tkach *et al*., 2016).

This study revealed the extent of the full size of the *E. miyagawai* mitochondrial genome highlighting the extensive NCR region. The actual length of the NCR was validated by a double-targeted multiplex-amplification and the continuous assembly of sequences achieved in the contigs by the long-read sequencing that spanned the whole region, revealing its multiple repetitive units. The long and short repetitive sequences, and complicated secondary structures of the NCR made it difficult to obtain the whole mtDNA for a species using conventional Sanger-sequencing (Kinkar *et al*., 2019, 2020; Le *et al*., 2022). The PacBio single-molecule real-time sequencing of *E. miyagawai* applied in this study yielded the much longer NCR in mtDNA and was shown to be different in length to those found in the Hunan and HLJ Chinese isolates which had been acquired by the Sanger-sequencing (Fu *et al*., 2019; Li *et al*., 2019). However, long read sequencing has also shown variation in NCR length to occur naturally within species of several Platyhelminthes such as *Echinococcus granulosus* genotype G1 (Kinkar *et al*., 2019), *Clonorchis sinensis* of South Korean origins (Kinkar *et al*., 2020), *Schistosoma bovis* (Oey *et al*., 2019*a*), *Paragonimus s. miyazakii* (Le *et al*., 2022), *Paragonimus westermani* Indian isolate IND2009 (Oey *et al*., 2019*b*), *Echinostoma revolutum* (Le *et al*., 2020) and *Artyfechinostomum malayanum* (Pham *et al*., 2022).

The base composition of A, T, G and C, as well as the skewness values of AT and GC content for PCGs, MRGs and NCR, indicated the use of T was more frequent than A, and G was more frequent than C. Within the *Echinostoma miyagawai* strains, the nucleotide divergence for the individual genes was under 1.57% and for the concatenated genes, PCGs, was between 0.58–0.69%, which is less than 2%, and is of a common value to be reported as a threshold of the intra-specific divergence for species delimitation (Jones *et al*., 2020). The genomic characteristics of the mitogenomic genes and genomes were shown to be highly similar within *E. miyagawai* species and close to *E. revolutum*, *E. paraensei* and *E. caproni*. The genetic closeness indicated the morphological status of the valid species belonging to the 37-collar-spined ‘*E. revolutum*” group (Kostadinova *et al*., 2000*a*; Georgieva *et al*., 2014; Le *et al*., 2020; Chai *et al*., 2021).

It is worth highlighting that the long-range PCR enrichment of mtDNA may have some disadvantages especially when attempting to capture the NCR and amplify a region with high frequency of repeat elements. In some species long PCR may skip complex regions and produce truncated resultant fragments. This has been overcome by using multiple target binding primers situated in the genes which flank the NCR in combination with high-fidelity proof reading *Taq* polymerase to increase the possibility of amplification through the complex region. This enrichment approach, in combination with targeted NGS sequencing, has successfully enabled the sequencing of the complete mitochondrial genomes of *Artyfechinostomum malayanum* (Pham *et al*., 2022), for *Paragonimus s. miyazakii* (Le *et al*., 2022), and for *E. miyagawai* in this study. Interestingly, intra species variation has been seen, with mitochondrial NCR length variation being seen between two isolates of Indian *P. westermani* that were sequenced using NGS (Biswal *et al*., 2014; Oey *et al*., 2019*b*). However, one of these isolates did undergo enrichment with long PCR to capture the NCR and the other was sequenced from a standard genomic DNA extract (Biswal *et al*., 2014; Oey *et al*., 2019*b*). It is important to note that the length variation could indeed be a consequence of the inability of NGS methods to capture the full NCR regions effectively without the PCR enrichment approach rather than a true inter isolate polymorphism. If the mitochondrial control region is essential to address certain biological questions, then mtDNA enrichment with targeted multiplex NGS sequencing should be applied, especially when validating NCR length between species and isolates.

The difference in length in the NCR between the three *E. miyagawai* geographical strains was a consequence of the numbers of the LRUs and SRUs (Fu *et al*., 2019; Li *et al*., 2019) and is seen in the other echinostomatids and trematodes. It should be noted that there were no fixed quantity of repeats and equal length for each in echinostomatids and trematodes to be found. The mitochondrial NCR region for *E. miyagawai* was shown to consist of two repetitive units the LRU and the SRU, a typical pattern seen in eukaryotic species (Bronstein *et al*., 2018). Similar pattern have been reported in *Clonorchis sinensis* which also raised questions about the functionality of the NCR or the ‘control’ regions within platyhelminth mitochondrial genomes, especially as expansive repetitive NCR with substantial size variation appears to be unusual within a species (Kinkar *et al*., 2020). It has been proposed that such tandem repeats units enhance replication and transcription efficiency, enabling the parasite to better withstand environmental pressures which change at each life cycle stage. Indeed, within the *E. miyagawai* typical promoter sequences containing TA(A)n, TATA and G(A)nT motifs were identified in both the LRU and the SRU, indicating that there are multiple sites to initiate transcription as have been seen in other invertebrate species (Tyagi *et al*., 2020). Similarly, palindromic sequences were also identified throughout both the LRU and the SRU, these are unique inverted repeats creating a hair pin structure acting as the recognition sites for DNA binding proteins involved in gene regulation (Arunkumar and Nagaraju, 2006). Analyses in this study also showed that several of these palindromic structures were conserved between echinostomatid species which could indicate that they could have some sort of selective advantage as has been suggested in other insects and nematodes (Arunkumar and Nagaraju, 2006). However, despite their importance in gene regulation many of the palindromes in *E. miyagawai* contained mismatches or indels in the stem portion of the hairpin indicating that these palindromic repeats could be in a state of deterioration as has been seen in other invertebrates and protists (Arunkumar and Nagaraju, 2006; Smith, 2020; Miyazawa *et al*., 2021). Such mutational decay has been shown to occur in small asexual populations allowing the accumulation of deleterious mutations because of Muller's Ratchet, and mitochondrial genomes have been shown to be particularly sensitive to the process owing to their uniparental inheritance, high mutation rates and lack of recombination. Studies in asexual wild populations of the nematode *Caenorhabditis briggsae* illustrated the accumulation of NCRs within the mitochondria and that compensatory mutations evolve to ensure functional gene regulation (Howe and Denver, 2008). Trematodes undergo clonal expansion within in the snail host increasing the chance for random mutational events occuring, which could have a significant impact on non-recombing regions of the genome, particular the mitochondria (Hammoud *et al*., 2022; Correia *et al*., 2023). <AQ: Please confirm the change of year from Hammoud *et al*. (2021) to Hammoud *et al*. (2022) in the text citation as per the reference list.>Over evolutionary time the repeat units could have accumulated as mutations owing to the effects on Muller's Ratchet acting on the asexual stages of the parasites, and because of the lack of recombination in the mitochondrial genome there would be a lack of proofreading to resolve mutations as seen in other organisms that undergo asexual reproduction (Garg and Martin, 2016). Mutations within NCR could indeed affect gene regulations, impacting on the efficacy of transcription factors binding to promotor regions. At least one of the units within the repeat could be functional and the tandem repeated units may provide some adaptive advantage as part of compensatory evolutionary response against the impact of Muller's Ratchet as seen elsewhere (Garg and Martin, 2016; Howe and Denver, 2008). However, in order to disentangle the function and evolution of these repeat units in trematodes considerable further work in laboratory and wild populations is required.

The present study provides the longest whole mitogenome of *Echinostoma miyagawai*, obtained by long-read sequencing of long-range PCR amplicons using the PacBio system, giving the mitogenomic data for the characterization of the polymorphisms and genetic features of the echinostomatid congeners. The mtDNA of this strain was found to possess a very long NCR (6 kb) with multiple tandem repeats, a higher number than those found in the other 2 Chinese strains. As previously reported, targeted multiplexed long-read sequencing proved to be the most advanced and effective approach for achieving the realistic extent of the mtDNA for a trematode species. However, it remains unclear to the extent that the mitochondrial NCR is polymorphic in terms of tandem repeats and if this represents true polymorphisms between different geographical isolates of echinostomatids as well as other trematodes

Fully characterized mitogenomes of additional members of the Echinostomatidae, are necessary. The mitogenomic datasets and the targeted-multiplexed long-read sequencing approach presented in this study will be useful for studies of taxonomic, evolutionary, and population genetics, and applicable to other taxa in the suborder Echinostomata and the class Trematoda.

## Supporting information

Pham et al. supplementary material Pham et al. supplementary material

## Data Availability

All DNA sequences used in this study are available on GenBank and accession numbers are provided through out the manuscript

## Supplementary material

The supplementary material for this article can be found at https://doi.org/10.1017/S0031182024001422.
