# Supplementary material for: Mitogenomics of the zoonotic parasite Echinostoma miyagawai and insights into the evolution of tandem repeat regions within the mitochondrial non-coding control region
Source: Parasitology. 2024 Nov 14;151(14):1543–54. doi: 10.1017/S0031182024001422 (PMC12052430; doi:10.1017/S0031182024001422)
Supplement: Pham et al. supplementary material [file S0031182024001422sup001.docx]

**Supplementary Material:**

**Mitogenomics of the zoonotic parasite *Echinostoma miyagawai* and insights into the evolution of tandem repeat regions within the mitochondrial non-coding control region**

Linh Thi Khanh Pham^1,2^, Dong Van Quyen^2^, Weerachai Saijuntha^3^, Huong Thi Thanh Doan^1,4^, Thanh Hoa Le^1,4^ and Scott P. Lawton^5^*

This document contains the supplementary information associated with analysed data on the mitochondrial genomes of *Echinostoma miyagawai* and its comparisons to other trematode species.

**Supplementary Table 1.** List of trematode-universal and specific primers for long-PCR used for enrichment of the mitochondrial genome of *Echinostoma miyagawai* and amplicons for next-generation sequencing

|  | **Primer pair** | **Length of amplicons** | **Spanning**  **region**  **(from/to)** | **Position in**  **the genome** | **Primer sequence**  **(5’ to 3’)** |
| --- | --- | --- | --- | --- | --- |
| 1 | ECH3F-JNAD1R | 5.7 kb | *cox*3–*nad*1 | 1–5682 | ECH3F: ATGAKTTGRTTGCCWATRTATAAAGC  JNAD1R: ATACACATAAAACAGGCCTC  ECHN4F: AGTTTGATTGGTATAGTTGGGG  ECHN2F: CTTGTTGGTGTCATATGATGC  URNSR: TACCATGTTACGACTTACCACA  UNI16R: TCTCGGGGTCTTTCCGTCT  UNI16F: TGGCCGCAGTATHTTGACTGTGC  GLYR: ACKAGACCHCYGACTTGGAAAGAC  URNLF: AGCCAGGTTGGTTCTTATCTAT  EMY5F: AGTTTCGTTCTATCGTTGGGAGGT  EMY3R: ACTACAAATTCTTCCTCCCTGT  EHC5F: TGTTTCTTTYTATCGTTGGGAGGT  EHC3R: CCCCCACACCAAAAATAACTCAA |
| 2 | ECHN4F-JNAD1R | 3.6 kb | *nad*4–*nad*1 | 2072–5682 |  |
| 3 | ECHN2F-URNSR | 5.7 kb | *nad*2–*rrn*S | 4603–10280 |  |
| 4 | ECHN2F-UNI16R | 4.6 kb | *nad*2–*rrn*L | 4603–9216 |  |
| 5 | UNI16F-GLYR | 4.4 kb | *rrn*L–*trn*G | 9050–13395 |  |
| 6 | URNLF-GLYR | 4.0 kb | *rrn*L–*trn*G | 9455–13395 |  |
| 7 | EMY5F-EMY3R* | 6.5 kb | *nad*5–*cox*3 | 13177-248 |  |
| 8 | EHC5F-EHC3R* | 7.0 kb | *nad*5–*cyt*B | 13200–789 |  |

*Primer pairs 7 and 8 were used for amplifying the NCR for validation of the length and for NGS sequencing

**Supplementary Table 2.** List of species providing information for the available mitochondrial genome in the suborder Echinostomata and other trematodes used in this study for sequence comparative and phylogenetic analyses

|  | **Family/Species/ Strains** | **Strains or designed** | **Country**  **of**  **collection** | **GenBank** | **mtDNA as**  **reported (bp)** | **mt**  **DNA** | **PCGs** | **MRGs** | **Reference**  **(if any)** |
| --- | --- | --- | --- | --- | --- | --- | --- | --- | --- |
|  | **Suborder Echinostomata** |  |  |  |  |  |  |  |  |
|  | **Echinostomatidae (14/12)** |  |  |  |  |  |  |  |  |
| 1 | *Artyfechinostomum malayanum* | (EMI3) | Thailand | OK509083 | 17175 | 13408 | 10131 | 1725 | Pham *et al*. (2022) |
| 2 | *Artyfechinostomum sufrartyfex* | (Shillong) | India | KY548763 | 14567 | 13409 | 10131 | 1728 | GenBank |
| 3 | *Echinostoma caproni* | (SAMEA) | Egypt | AP017706 | 14150 | 13293 | 10128 | 1709 | GenBank |
| 4 | *Echinostoma miyagawai* | (RED11) | Thailand | OP326312 | 19417 | 13324 | 10128 | 1725 | This study |
| 5 | *Echinostoma miyagawai* | (Hunan)* | China | MN116740 | 14460 | 13320 | 10128 | 1724 | Fu *et al*. (2019) |
| 6 | *Echinostoma miyagawai* | (HLJ)* | China | MH393928 | 14410 | 13321 | 10128 | 1763 | Li *et al*. (2019) |
| 7 | *Echinostoma paraensei* | n/a | n/a | KT008005 | 20298 | 13319 | 10128 | 1748 | GenBank |
| 8 | *Echinostoma revolutum* | (MSD15) | Thailand | MN496162 | 17030 | 13326 | 10134 | 1733 | Le *et al*. (2020) |
| 9 | *Echinostoma revolutum*? | (GD) | China | MN116706 | 15714 | 13282 | 10149 | 1754 | Ran *et al*. (2020) |
| 10 | *Echinostoma* sp. | (JM-2019) | China | MH212284 | 15283 | 13257 | 10122 | 1726 | GenBank |
| 11 | Echinostomatidae sp.  CA-2021 | (PE4) | United States | MK264774 | 14426 | 13319 | 10143 | 1727 | GenBank |
| 12 | Echinostomatidae sp.  MSB para 30070 | (A19) | United States | MN822299 | 13985 | 13346 | 10128 | 1732 | GenBank |
| 13 | *Hypoderaeum conoideum* | (Hubei) | China | KM111525 | 14180 | 13361 | 10116 | 1730 | Yang *et al*. (2015) |
| 14 | *Echinoparyphium aconiatum* | (Chany) | Russia | ON644993 | 14865 | 13377 | 10113 | 1730 | Gacad *et al*. (2023) |
|  | **Cyclocoelidae (3/3)** |  |  |  |  |  |  |  |  |
| 15 | *Morishitium polonicum* | (Laojun) | China | OP930879 | 14083 | 13337 | 10137 | 1720 | Liu *et al*. (2023) |
| 16 | *Tracheophilus cymbius* | (HLJ) | China | MK355447 | 13760 | 13458 | 10152 | 1745 | Li *et al*. (2019) |
| 17 | *Uvitellina* sp. SSS2019 | (SSS2019) | Pakistan | MK227160 | 14217 | 13705 | 10200 | 1751 | Suleman *et al*. (2019) |
|  | **Echinochasmidae (1/1)** |  |  |  |  |  |  |  |  |
| 18 | *Echinochasmus japonicus* | (PT) | Vietnam | KP844722 | 15865 | 13378 | 10143 | 1748 | Le *et al*. (2016) |
|  | **Fasciolidae (7/6)** |  |  |  |  |  |  |  |  |
| 19 | *Fasciola gigantica* | (GX) | China | KF543342 | 14478 | 13309 | 10107 | 1755 | Liu *et al*. (2014) |
| 21 | *Fasciola hepatica* | (GL) | Australia | AF216697 | 14462 | 13305 | 10104 | 1755 | Le *et al*. (2001) |

mtDNA: the entire mitochondrial genome; mtDNA*: the coding mitochondrial genome (5’ terminus of *cox*3 to 3’ terminus of *nad*5); PCGs: protein-coding genes; MRGs: mitoribosomal genes; *the length of the *Echinostoma miyagawai* Hunan (Hunan strain; MN116740) has been corrected from 14,468 bp to 14,460 bp, and HLJ (Heilongjiang strain; MH393928) from 14,416 bp to 14,410 bp. The numbers in a bracket indicate the number of isolates and species in that family used for the genetic and phylogenetic analyses.

**Supplementary Table 3:**

Locations of genes and other features in the mitochondrial genomes of *Echinostoma miyagawai* (EMIY-RED11-TH, 19,417 bp, GenBank OP326312; and strain EMIY-Hunan-CN, 14,460 bp, GenBank MN116740)

| **Gene/**  **Region** | **Position**  **(5’ > 3’)** | **Characteristics**  **[bp/aa(start/stop)]**  **and regions** | **Int. seq.**  **(bp)** | **tRNA**  **anti-codon** | **Position**  **(5’ > 3’)** | **Characteristics**  **[bp/aa(start/stop)]**  **and regions** | **Int. seq.**  **(bp)** |
| --- | --- | --- | --- | --- | --- | --- | --- |
|  | *Echinostoma miyagawai*  (EMIY-RED11-TH, Thailand, OP326312) | | |  | *Echinostoma miyagawai*  (EMIY-Hunan-CN, China, MN116740) | | |
| *cox*3 | 1–645 | 645/214/(ATG/TAA) | ­­­+3 |  | 1–645 | 645/214/(ATG/TAA) | ­­­+3 |
| tRNA^His^ (*trn*H) | 649–713 | 65 | +2 | GTG | 649–713 | 65 | +2 |
| *cob* | 716–1825 | 1110/369/(ATG/TAG) | 0 |  | 716–1825 | 1110/369/(ATG/TAG) | +0 |
| *nad*4L | 1826–2098 | 273/90/(ATG/TAG) | –40 |  | 1826–2098 | 273/90/(ATG/TAG) | –40 |
| *nad*4 | 2059–3342 | 1284/427/(ATG/TAG) | +4 |  | 2059–3342 | 1284/427/(GTG/TAA) | +4 |
| tRNA^Gln^ (*trn*Q) | 3347–3410 | 64 | +8 | TTG | 3347–3410 | 64 | +8 |
| tRNA^Phe^ (*trn*F) | 3419–3484 | 66 | +33 | GAA | 3419–3484 | 66 | +33 |
| tRNA^Met^ (*trn*M) | 3518–3583 | 66 | +3 | CAT | 3518–3583 | 66 | +3 |
| *atp*6 | 3587–4105 | 519/172/(ATG/TAG) | +7 |  | 3587–4105 | 519/172/(ATG/TAG) | +7 |
| *nad*2 | 4113–4982 | 870/289/(ATG/TAG) | +4 |  | 4113–4982 | 870/289/(ATG/TAG) | +4 |
| tRNA^Val^ (*trn*V) | 4987–5050 | 64 | +24 | TAC | 4987–5040 | 54 | +34 |
| tRNA^Ala^ (*trn*A) | 5075–5142 | 68 | +4 | TGC | 5074–5142 | 68 | +4 |
| tRNA^Asp^ (*trn*D) | 5147–5212 | 66 | 0 | GTC | 5147–5212 | 66 | 0 |
| *nad*1 | 5213–6115 | 903/300/(GTG/TAG) | +6 |  | 5213–6115 | 903/301/(ATG/TAG) | +6 |
| tRNA^Asn^ (*trn*N) | 6122–6188 | 67 | +4 | GTT | 6122–6188 | 67 | +4 |
| tRNA^Pro^ (*trn*P) | 6193–6261 | 69 | +1 | TGG | 6193–6261 | 69 | +1 |
| ttRNA^Ile^ (*trn*I) | 6263–6324 | 62 | +9 | GAT | 6263–6324 | 62 | +8 |
| tRNA^Lys^ (*trn*K) | 6334–6402 | 69 | +4 | CTT | 6333–6402 | 69 | +4 |
| *nad*3 | 6407–6763 | 357/118/(ATG/TAG) | +3 |  | 6406–6762 | 357/118/(ATG/TAA) | +3 |
| tRNA^Ser1(AGN)*^ (*trn*S_1_) | 6767–6826 | 60 | +4 | GCT | 6766–6825 | 60 | +4 |
| tRNA^Trp^ (*trn*W) | 6831–6896 | 66 | +3 | TCA | 6830–6895 | 66 | +3 |
| *cox*1 | 6900–8438 | 1539/512/(GTG/TAA) | +35 |  | 6899–8437 | 1539/512/(ATG/TAG) | +35 |
| tRNA^Thr^ (*trn*T) | 8474–8543 | 70 | 0 | TGT | 8473–8542 | 70 | 0 |
| *rrn*L (16S) | 8544–9518 | 975 | 0 |  | 8543–9517 | 975 | 0 |
| tRNA^Cys^ (*trn*C) | 9519–9585 | 67 | 0 | GCA | 9518–9584 | 67 | 0 |
| *rrn*S (12S) | 9586–10335 | 750 | 0 |  | 9585–10333 | 749 | 0 |
| *cox*2 | 10336–10944 | 609/202/(ATG/TAG) | +11 |  | 10334–10942 | 609/202/(ATG/TAG) | +11 |
| *nad*6 | 10956–11408 | 453/150/(ATG/TAG) | 0 |  | 10954–11406 | 453/150/(ATG/TAG) | 0 |
| tRNA^Tyr^ (*trn*Y) | 11409–11477 | 68 | 0 | GTA | 11407–11473 | 67 | 0 |
| tRNA^Leu1(CUN)^ (*trn*L_1_) | 11478–11542 | 65 | –3 | TAG | 11474–11538 | 65 | -2 |
| tRNA^Ser2(UCN)*^ (*trn*S_2_) | 11540–11604 | 65 | +27 | TGA | 11537–11599 | 65 | +28 |
| tRNA^Leu2(UUR)^ (*trn*L_2_) | 11632–11694 | 63 | 0 | TAA | 11628–11690 | 63 | 0 |
| tRNA^Arg^ (*trn*R) | 11695–11758 | 64 | 0 | TCG | 11691–11754 | 64 | 0 |
| *nad*5 | 11759–13324 | 1566/521/(GTG/TAG) | +19 |  | 11755–13320 | 1566/521/(ATG/TAG) | +19 |
| tRNA^Gly^ (*trn*G) | 13344–13409 | 66 | +9 | TCC | 13340–13405 | 66 | +9 |
| tRNA^Glu^(*trn*E) | 13418–13482 | 64 | +22 | TTC | 13415–13478 | 64 | +8 |
| **NCR region** | 13483-19417 | 5935 |  |  | 13481–14460 | 982 |  |
| **LRU region** | 13505–18392 | 48888 |  |  | 13487–14437 | 951 |  |
| LRU1 | 13506–13824 | 319 | 0 |  | 13488–13806 | 319 | 0 |
| LRU2 | 13825–14143 | 319 | 0 |  | 13807–14125 | 319 | 0 |
| LRU3 | 14144–14462 | 319 | 0 |  | 14126–14437# | 312 | 0 |
| LRU4 | 14463–14781 | 319 | 0 |  |  |  |  |
| LRU5 | 14782–15100 | 319 | 0 |  |  |  |  |
| LRU6 | 15101–15419 | 319 | 0 |  |  |  |  |
| LRU7 | 15420–15738 | 319 | 0 |  |  |  |  |
| LRU8 | 15739–16057 | 319 | 0 |  |  |  |  |
| LRU9 | 16058–16376 | 319 | 0 |  |  |  |  |
| LRU10 | 16377–16695 | 319 | 0 |  |  |  |  |
| LRU11 | 16696–17014 | 319 | 0 |  |  |  |  |
| LRU12 | 17015–17333 | 319 | 0 |  |  |  |  |
| LRU13 | 17334–17652 | 319 | 0 |  |  |  |  |
| LRU14 | 17653–17971 | 319 | 0 |  |  |  |  |
| LRU15 | 17972–18290 | 319 | 0 |  |  |  |  |
| LRU15.3# | 18291–18392 | 102 | 0 |  |  |  |  |
| Junction seq. | 18393–18395 | 3 | 0 |  |  |  |  |
| **SRU Region** | 18396–19412 | 1017 |  |  |  |  |  |
| SRU1 | 18396–18608 | 213 | 0 |  |  |  |  |
| SRU2 | 18609–18821 | 213 | 0 |  |  |  |  |
| SRU3 | 18822–19034 | 213 | 0 |  |  |  |  |
| SRU4 | 19035–19247 | 213 | 0 |  |  |  |  |
| SRU4.8# | 19248–19412 | 165 | 0 |  |  |  |  |
| Uni. seq. | 19413–**19417** | 5 | 0 |  | 14438–**14460** | 23 | 0 |

bp: base pair; aa: amino acid; start: start codon; stop: stop codon; Int. seq.: intergenic sequence (+. number of nucleotides before start of following gene; –, number of nucleotides overlapping with following gene); Junction seq.: sequence connecting the last LRU and first SRU; Uni. seq.: sequence between last SRU and *cox*3; *asterisk: tRNAs lacking DHU-arm. RU#: imperfect repeat; unit for Emiy-RED11-TH (LRU15.3# and SRU4.8#) and for Emiy-Hunan-CN (LRU2.99#, position: 14126–14437#).

**Supplementary Table 4.** Base composition and skew/skewness value for AT and GC of the protein-coding genes (PCGs), mito-ribosomal genes (MRGs), and the coding region (abbreviated as mtDNA*) of the mitogenomes of *Echinostoma miyagawai* and other echinostomatid members of the family Echinostomatidae

| **Species/Strains** | **Sequence** | **Length**  **(nt)** | **A**  **(%)** | **T**  **(%)** | **G**  **(%)** | **C**  **(%)** | **A+T**  **(%)** | **AT-skew** | **G+C**  **(%)** | **GC-skew** |
| --- | --- | --- | --- | --- | --- | --- | --- | --- | --- | --- |
| ***Echinostoma miyagawai***  (Emiya-RED11-TH)  (OP326312) | PCGs | 10128 | 18.05 | 47.60 | 24.22 | 10.13 | 65.65 | –0.450 | 34.35 | 0.410 |
|  | MRGs | 1725 | 25.68 | 37.80 | 23.59 | 12.93 | 63.48 | –0.191 | 36.52 | 0.292 |
|  | mtDNA* | 13324 | 19.70 | 45.38 | 24.26 | 10.66 | 65.08 | –0.395 | 34.92 | 0.389 |
| ***Echinostoma miyagawai***  (Emiya-Hunan-CN)  (MN116740) | PCGs | 10128 | 18.20 | 47.65 | 24.07 | 10.08 | 65.85 | –0.447 | 34.15 | 0.410 |
|  | MRGs | 1724 | 25.75 | 37.94 | 23.49 | 12.82 | 63.72 | –0.191 | 36.31 | 0.294 |
|  | mtDNA* | 13320 | 20.18 | 45.43 | 23.82 | 10.57 | 65.61 | –0.385 | 34.39 | 0.385 |
| ***Echinostoma miyagawai***  (Emiya-HLJ-CN)  (MH393928) | PCGs | 10128 | 18.17 | 47.50 | 24.12 | 10.21 | 65.67 | –0.447 | 34.33 | 0.405 |
|  | MRGs | 1763 | 25.98 | 37.61 | 23.60 | 12.82 | 63.59 | –0.183 | 36.41 | 0.296 |
|  | mtDNA* | 13321 | 19.85 | 45.24 | 24.22 | 10.68 | 65.09 | –0.390 | 34.91 | 0.388 |
| ***Echinostoma caproni***  (Ecapr-SAMEA-EG)  (AP017706) | PCGs | 10128 | 17.34 | 47.82 | 24.79 | 10.05 | 65.16 | –0.468 | 34.84 | 0.423 |
|  | MRGs | 1709 | 25.34 | 36.63 | 24.40 | 13.63 | 61.97 | –0.182 | 38.03 | 0.283 |
|  | mtDNA* | 13293 | 19.05 | 45.45 | 24.81 | 10.69 | 64.50 | –0.409 | 35.50 | 0.398 |
| ***Echinostoma paraensei***  (Epar)  (KT008005) | PCGs | 10128 | 18.04 | 47.57 | 24.13 | 10.26 | 65.61 | –0.450 | 34.39 | 0.403 |
|  | MRGs | 1748 | 25.92 | 37.76 | 23.68 | 12.64 | 63.68 | –0.186 | 36.32 | 0.304 |
|  | mtDNA* | 13319 | 19.81 | 45.42 | 24.12 | 10.66 | 65.23 | –0.393 | 34.77 | 0.387 |
| ***Echinostoma revolutum***  (Erevo-MSD15-TH)  (MN496162) | PCGs | 10134 | 18.81 | 47.40 | 23.50 | 10.29 | 66.21 | –0.432 | 33.79 | 0.391 |
|  | MRGs | 1733 | 25.74 | 36.99 | 23.77 | 13.50 | 62.73 | –0.179 | 37.27 | 0.276 |
|  | mtDNA* | 13326 | 20.35 | 45.21 | 23.60 | 10.84 | 65.56 | –0.379 | 34.44 | 0.371 |
| ***Echinostoma revolutum* (?)**  (Erev-GD-CN)  (MN116706) | PCGs | 10113 | 16.24 | 46.60 | 26.78 | 10.39 | 62.84 | –0.483 | 37.17 | 0.441 |
|  | MRGs | 1754 | 24.57 | 35.12 | 26.91 | 13.40 | 59.69 | –0.177 | 40.31 | 0.335 |
|  | mtDNA* | 13282 | 18.06 | 44.19 | 26.78 | 10.97 | 62.25 | –0.420 | 37.75 | 0.419 |
| ***Echinostoma* sp.**  (Ech-JM-2019-CN)  (MH212284) | PCGs | 10122 | 16.47 | 46.46 | 26.66 | 10.40 | 62.93 | –0.477 | 37.07 | 0.439 |
|  | MRGs | 1726 | 24.51 | 35.17 | 26.94 | 13.38 | 59.68 | –0.179 | 40.32 | 0.336 |
|  | mtDNA* | 13257 | 18.25 | 44.11 | 26.70 | 10.95 | 62.36 | –0.395 | 37.89 | 0.416 |
| **Echinostomatidae sp. CA-2021**  (EchCA2021-PE4-US)  (MK264774) | PCGs | 10143 | 17.50 | 45.81 | 26.19 | 10.50 | 63.31 | –0.447 | 36.69 | 0.428 |
|  | MRGs | 1727 | 25.25 | 34.80 | 26.40 | 13.55 | 60.05 | –0.159 | 39.95 | 0.322 |
|  | mtDNA* | 13319 | 19.18 | 43.37 | 26.32 | 11.14 | 62.55 | –0.387 | 37.45 | 0.405 |
| **Echinostomatidae sp. MSB para 30070** (EchMSB-A19-US) (MN822299) | PCGs | 10128 | 18.32 | 45.71 | 25.25 | 10.72 | 64.03 | –0.428 | 35.97 | 0.404 |
|  | MRGs | 1732 | 26.21 | 35.05 | 25.58 | 13.16 | 61.26 | –0.144 | 38.74 | 0.321 |
|  | mtDNA* | 13346 | 20.10 | 43.29 | 25.39 | 11.22 | 63.39 | –0.366 | 36.61 | 0.387 |
| ***Artyfechinostomum malayanum***  (Amala-EMI3-TH)  (OK509083) | PCGs | 10131 | 17.08 | 46.32 | 26.37 | 10.23 | 63.40 | –0.461 | 36.60 | 0.441 |
|  | MRGs | 1725 | 24.58 | 36.93 | 25.74 | 12.75 | 61.51 | –0.201 | 38.49 | 0.337 |
|  | mtDNA* | 13408 | 18.78 | 44.10 | 26.48 | 10.64 | 62.88 | –0.403 | 37.12 | 0.427 |
| ***Artyfechinostomum sufrartyfex***  (Asufr-Shillong-IN)  (KY548763) | PCGs | 10131 | 16.99 | 46.21 | 26.53 | 10.27 | 63.20 | –0.462 | 36.80 | 0.442 |
|  | MRGs | 1728 | 24.71 | 37.09 | 25.58 | 12.62 | 61.80 | –0.200 | 38.20 | 0.339 |
|  | mtDNA* | 13409 | 18.73 | 44.03 | 26.57 | 10.66 | 62.76 | –0.403 | 37.24 | 0.427 |
| ***Echinoparyphium aconiatum***  (Eacon-Chany-RU)  (ON644993) | PCGs | 10113 | 19.05 | 46.02 | 24.39 | 10.53 | 65.07 | –0.414 | 34.93 | 0.397 |
|  | MRGs | 1730 | 26.18 | 36.36 | 24.51 | 12.95 | 62.54 | –0.163 | 37.46 | 0.309 |
|  | mtDNA* | 13377 | 20.74 | 43.72 | 24.56 | 10.99 | 64.46 | –0.357 | 35.54 | 0.382 |
| ***Hypoderaeum conoideum***  (Hcono-Hubei-CN)  (KM111525) | PCGs | 10116 | 16.84 | 45.25 | 26.96 | 10.95 | 62.09 | –0.458 | 37.91 | 0.422 |
|  | MRGs | 1727 | 25.13 | 34.57 | 26.64 | 13.67 | 59.70 | –0.158 | 40.30 | 0.322 |
|  | mtDNA* | 13361 | 18.64 | 42.92 | 27.00 | 11.44 | 61.56 | –0.394 | 38.44 | 0.405 |

Information for strains and/or species is given in **Table S2**; their strain abbreviations and GenBank accession numbers are given in bracket after the taonomic name; PCGs: protein-coding genes; MRGs: mitoribosomal genes; mtDNA*: mitochondrial coding nucleotide sequence (from 5’ terminus of *cox*3 to 3’ terminus of *nad*5). *Echinostoma revolutum* (?): This is reported as *E. revolutum* (Ran *et al*., 2020) but may be a member of a “cryptic” genus.


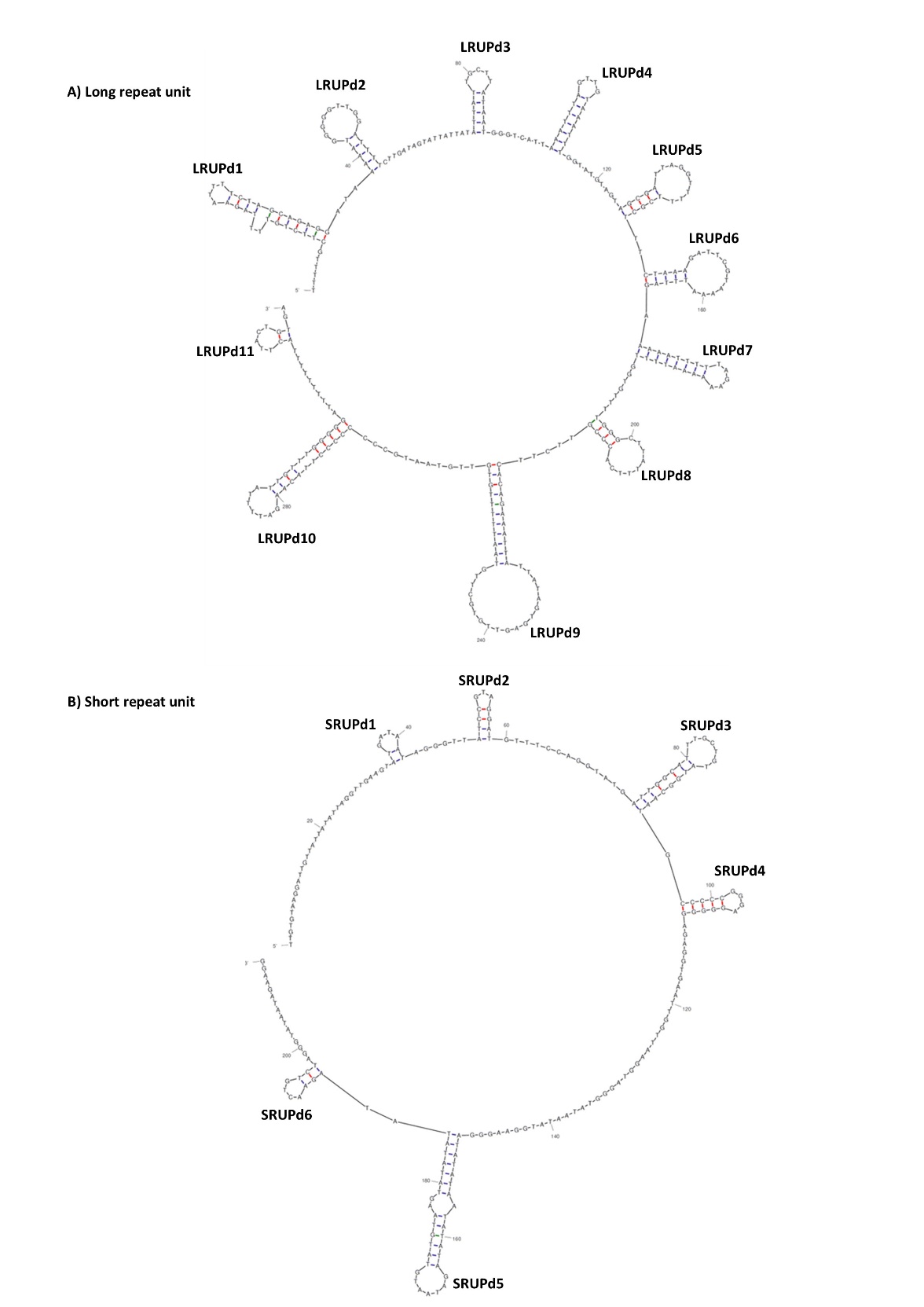


**Supplementary Figure 1: Predicted palindromic sequence sites within the tandem repeat units of the *Echinostoma miyagawai* mitochondrial control region.** Where A) illustrates the 11 palindromic sites that identified across the long repeat unit and B) represents the six palindromic sites across the short repeat unit. Blue dashes represent purine to pyrimidine bonds AT, red dashes represent purine to pyrimidine bonds GC, green dashes represent forced purine to pyrimidine bonds GT, and blanks represent the same bases.
